# Asymptomatic *Helicobacter Pylori* Infection in Preschool Children and Young Women Does Not Predict Iron Bioavailability from Iron-Fortified Foods

**DOI:** 10.3390/nu11092093

**Published:** 2019-09-04

**Authors:** Simone Buerkli, Ndèye Fatou Ndiaye, Colin I. Cercamondi, Isabelle Herter-Aeberli, Diego Moretti, Michael B. Zimmermann

**Affiliations:** 1 Laboratory of Human Nutrition, Institute of Food Nutrition and Health, ETH Zurich, 8092 Zurich, Switzerland; 2 Laboratoire de Nutrition, Département de Biologie Animale, Faculté des Sciences et Techniques, Université Cheikh Anta Diop de Dakar, 5005 Dakar-Fann, Senegal

**Keywords:** *Helicobacter pylori*, *H. pylori*, asymptomatic *H. pylori* infection, iron absorption, iron bioavailability, fortification, biofortification, preschool children, women of reproductive age

## Abstract

*Helicobacter pylori* infection is common in low-income countries. It has been associated with iron deficiency and reduced efficacy of iron supplementation. Whether *H. pylori* infection affects iron absorption from fortified and biofortified foods is unclear. Our objective was to assess whether asymptomatic *H. pylori* infection predicts dietary iron bioavailability in women and children, two main target groups of iron fortification programs. We did a pooled analysis of studies in women of reproductive age and preschool children that were conducted in Benin, Senegal and Haiti using stable iron isotope tracers to measure erythrocyte iron incorporation. We used mixed models to assess whether asymptomatic *H. pylori* infection predicted fractional iron absorption from ferrous sulfate, ferrous fumarate or NaFeEDTA, controlling for age, hemoglobin, iron status (serum ferritin), inflammation (C-reactive protein), and test meal. The analysis included 213 iron bioavailability measurements from 80 women and 235 measurements from 90 children; 51.3% of women and 54.4% of children were seropositive for *H. pylori*. In both women and children, hemoglobin (Hb), serum ferritin (SF), and C-reactive protein (CRP) did not differ between the seropositive and seronegative groups. Geometric mean (95% CI) fractional iron absorption (%), adjusted for SF, was 8.97% (7.64, 10.54) and 6.06% (4.80, 7.67) in *H. pylori* positive and negative women (*p* = 0.274), and 9.02% (7.68, 10.59) and 7.44% (6.01, 9.20) in *H. pylori* positive and negative children (*p* = 0.479). Our data suggest asymptomatic *H. pylori* infection does not predict fractional iron absorption from iron fortificants given to preschool children or young women in low-income settings.

## 1. Introduction

*Helicobacter pylori* (*H. pylori*) is a common colonizer of the human gastric mucus [1]. It is estimated that ≈50% of the global population may be infected [2], and prevalence is higher in low income countries, ranging from 65% in adults from Thailand [3] and Ethiopia [4] to 85% in mothers from Bangladesh [5]. Infection with *H. pylori* usually begins during childhood and may have lifelong persistence if not treated; in Bangladesh prevalence range from 47% in children below the age of 2 [5], 60% in children less than 5 years-old [6], up to 93% prevalence in children below the age of 15 [7]. Although infection with *H. pylori* is a major risk factor for chronic gastritis, peptic ulcer disease, and gastric cancer, the majority of cases remain asymptomatic [2].

A systematic review of studies conducted in adults and children, concluded that *H. pylori* infection increases the risk for low iron status. The meta-analysis reported a 1.33 odds ratio for iron deficiency (ID) among seropositive individuals, a 1.15 odds ratio for anemia, and a 1.72 odds ratio for iron deficiency anemia (IDA) [8]. A large cross-sectional study in China (*n* = 17791, mean age 45 ± 18 years old) reported a significantly higher prevalence of anemia in *H. pylori* infected individuals [9]. The clinical outcome of an *H. pylori* infection remains complex as an antral mucosa infection leads to increased gastric acid secretion. In contrast, infection of the corpus mucosa leads to decreased acid secretion, which is observed in the majority of the infected patients [10,11]. Further, gastric ascorbic acid secretion was reported to be significantly lower in *H. pylori* infected versus uninfected children [12]. Several studies have associated *H. pylori* infection with depleted iron stores; in symptomatic adults: Atrophic gastritis is associated with IDA, and lower hemoglobin (Hb) levels [13,14,15]. In symptomatic children, lower serum ferritin (SF), hemoglobin, serum iron and transferrin were associated with *H. pylori* infected children with hypochlorhydria [16,17].

Studies examining iron absorption during *H. pylori* infection have produced equivocal results. Two studies in adults with *H. pylori* infection reported impaired iron absorption from a test meal or an iron dose [18,19]. In contrast, in a study in Bangladeshi children, there was no significant difference in iron absorption before and after treatment of *H. pylori* infection, although the *H. pylori* positive children had impaired gastric acid production [20]. A subsequent study in Bangladeshi children reported no significant associations between *H. pylori* infection, iron deficiency, iron-deficiency anemia and iron absorption [21].

IDA is a major global public health problem in children and young women [22]. Iron fortification can be a cost-effective approach to prevent IDA [23,24,25], and fortification programs have been introduced in many low-income countries [26]. Whether *H. pylori* contributes to the high prevalence of IDA in children and young women and/or blunts the efficacy of iron fortification programs remains unclear. Therefore, our study aim was to assess whether asymptomatic *H. pylori* infection is associated with iron bioavailability from commonly-used iron food fortificants in preschool children and women of reproductive age. Our hypothesis was that seropositive *H. pylori* infection would be an independent predictor of lower iron bioavailability from iron fortificants in both of these age groups.

## 2. Materials and Methods

### 2.1. Subjects and Study Design

This was a retrospective pooled analysis of stable iron isotope absorption studies done in women and preschool children that were carried out in Benin [27,28,29], Haiti [30], and Senegal (N. Ndiaye, unpublished results) between 2010 and 2015. All studies had ethical approval from locally independent ethics committees and from ETH Zurich, Switzerland. Written informed consent was obtained from the subjects in the adult studies or the caregiver of the children in the pediatric studies. The studies were registered at clinicaltrials.gov (NCT01108939, NCT01321099, NCT01634932, NCT02096250, and NCT02437955).

All studies followed the same general design and used standardized methods to assess *H. pylori* infection and iron bioavailability. Subjects were recruited, screened for eligibility and infection with *H. pylori* was assessed at baseline. Inclusion criteria for all studies were: (1) Apparently healthy with no history of a diagnosed gastrointestinal disorder, or another chronic disease; (2) had not received a blood transfusion or had substantial blood loss within six months before the start of the study; (3) did not consume vitamin or mineral supplements within two weeks before the study start; (4) for women, not pregnant or lactating, and (5) for women, bodyweight < 65 kg. Included participants then consumed iron fortified test meals labelled with stable iron isotopes (^54^Fe, ^57^Fe, ^58^Fe, as ferrous sulfate, ferrous fumarate or ferrous sodium EDTA). All test meals were administered in a single-blind, randomized cross-over design. After a study-specific incorporation period (minimum of 14 days), a venous blood sample was taken to measure incorporation of the stable iron isotopes into erythrocytes.

**Figure 1:**
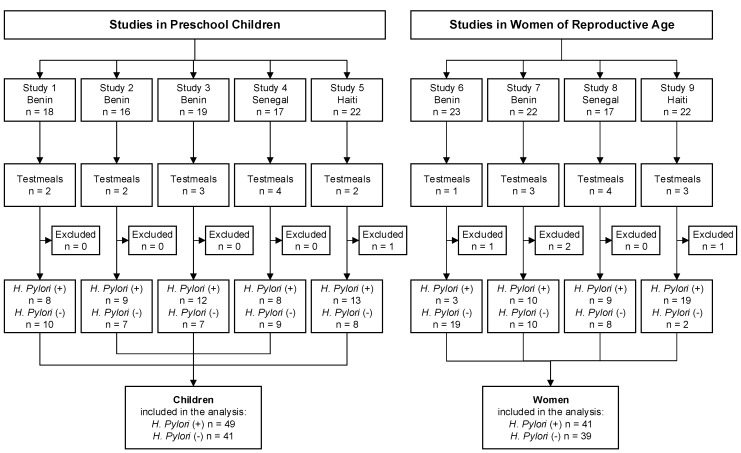
Study flow chart: Five iron absorption studies in preschool children and four iron absorption studies in women of reproductive age were conducted at different sites. Five participants were excluded from the analysis because *Helicobacter pylori* status was not assessed.

Table 1 gives an overview of the iron compounds and test meals of all studies, and Figure 1 the flow of participants. All of the studies, except the studies conducted in Senegal, have been previously described in detail. Studies 1 to 3 were done in children (*n* = 52) aged 1.5 to 3 years, at the local state hospital in Natitingou in Benin. All children were afebrile, had a negative malaria smear, had a bodyweight > 8.3 kg and weight-for-age z-score (WAZ) > −3. The children were divided into three studies, and all groups received a millet-based porridge with different iron compounds and absorption enhancers (ascorbic acid or phytase). (Table 1) [28]. Study 6 was done in Beninese women (*n* = 23), at the local state hospital in Natitingou and Toucountouna in Benin, aged 16 to 40 years without severe anemia (Hb > 8 g/dL), who received a labelled sorghum-based test meal (Table 1) during afebrile malaria. Following successful treatment of malaria and inflammation, participants received a second labeled test meal. The analysis in the current manuscript only includes the iron bioavailability data from the test meal that was administered after malaria treatment [27]. One participant from this study was excluded due to missing sample. Study 7 was done in Beninese women (*n* = 22), at the local state hospital in Natitingou, aged 17 to 35 years, who had a negative malaria smear and depleted iron stores (SF < 25 µg/L). They were given a millet-based test meal (Table 1) [29]. Two participants from this study were excluded due to missing samples. Studies 4 and 8 were done in Senegal, at the laboratory of nutrition at the University of Cheikh Anta Diop in Dakar. It included non-anemic (Hb > 11 g/dL) women (study 8, *n* = 17) and their preschool children (study 4, *n* = 17), aged 3 to 6 years; they received wheat bread test meals with a herbal tea (polyphenol-rich tea, inhibitor of iron absorption) or water, fortified with either ferrous sulfate or ferrous fumarate. One mother and one child from this study were excluded due to missing samples. Studies 5 and 9 were done in Haiti at the Ministry of Health in Port au Prince. It included mothers (study 9, *n* = 22) without severe anemia (Hb > 10 g/dL) and their preschool child (study 4, *n* = 22), aged 2.5 to 5 years, with weight-for-height (WHZ) and height-for-age z-score (HAZ) > −2; they were given a wheat bread-based test meal [30]. One participant of each study was excluded due to missing sample.

All studies used isotopically enriched elemental iron ^54^Fe, ^57^Fe, and ^58^Fe, which were purchased from Chemgas (Boulogne, France). The isotopically labeled iron compounds were produced as described in each study [27,28,29,30].

### 2.2. Laboratory Analysis

At recruitment, hemoglobin, serum ferritin, C-reactive protein (CRP), and anthropometrics were measured as previously described [27,28,29,30]. Anemia in women was defined as Hb < 120 g/L; in children ≥ 5 year-old, Hb < 115 g/L, and in children < 5 year-old, Hb < 110 g/L. ID in women was defined as SF < 15 µg/L; in children ≥ 5 y, SF < 1 5 µg/L; in children <5 y: SF < 12 µg/L. IDA in women and in children was defined as both Hb and SF below these cutoffs [31]. *H. pylori* infection was assessed from a baseline serum sample using a qualitative rapid immunochromatographic assay (rapid anti-*H. pylori* test, Rapid Labs Ltd., Essex, UK), to detect the presence of IgG antibodies anti-*H. pylori* in serum or plasma. This test has 86.7% relative sensitivity and 91% relative specificity, and an overall agreement of 89.8% compared to an ELISA assay [32]. The test was carried out according to the instructions of the manufacturer: One drop of thawed serum was placed on the sample well, and then one drop of sample diluent was added, the result was read after 15 minutes; the test was judged invalid if the control line did not appear. Iron bioavailability (erythrocyte iron incorporation) was calculated based on the shift in isotope ratios and the estimated amount of iron circulating in the body, with the use of the participants’ blood volume [33]. The calculations are based on the principles of isotope dilution, considering that iron isotopic labels were not monoisotopic [34,35,36]. Details have been previously described [27,28,29,30].

### 2.3. Statistical Analysis

To detect an inter-subject difference of 30% with a β of 0.8 and α of 0.05, a sample size of 33 per group was calculated to be sufficient. Data were analyzed in IBM SPSS statistics (version 23). For children under five years of age WHO Anthro (v3.2.2, 2011; WHO) and above five years WHO Anthro plus (v1.0.4, 2007; WHO) was used to calculate age-specific HAZ, WAZ, and WHZ. Adjustment of serum ferritin for inflammation (CRP) was performed as previously described [37]. Iron bioavailability was also standardized to an SF concentration of 40 µg/L, as described [38]. Statistical tests were performed on both unadjusted and adjusted iron bioavailability data. Data were tested for normal distribution by using the Kolmogorov-Smirnov test. If the data were not normally distributed, log-transformed data were used for analysis. Normally distributed data are presented as mean ± SD (WAZ, HAZ, WHZ) and not normally distributed data as geometric mean and 95% confidence interval (age, weight, height, Hb, SF, CRP, fractional iron absorption). Independent samples *t*-tests were performed to detect differences in iron status, and anthropometric measures between *H. pylori* positive versus negative groups. To detect differences in fractional iron absorption, a linear mixed model (LMM) analysis was performed with subject ID as random intercept, fractional iron absorption as dependent factor, *H. pylori* infection, the iron compound, the food matrix of the test meal and whether the meal was given with an enhancer or inhibitor of iron absorption as fixed factors. The same model was repeated with the SF corrected fractional iron absorption data. To describe predictors of fractional iron absorption in these populations, a backward linear regression was performed separately for children and women to find the minimal adequate model. Fractional iron absorption was set as dependent variable and independent variables were: *H. pylori* infection, the food matrix of the test meal, whether the test meal was given with an enhancer of inhibitor of iron absorption, the iron compound, gender (in the children’s model), age, Hb, the SF adjusted for CRP and the subject ID. Then an LMM analysis was performed with subjects’ ID defined as random intercept, fractional iron absorption as the dependent variable, and the variables from the minimal adequate model set as fixed factors. *p* values < 0.05 were considered as statistically significant.

## 3. Results

This analysis included data from 84 women and 91 children (Table 2); four women and one child were excluded from the analysis because *H. pylori* status could not be determined. Data on iron bioavailability from 11 different test meals were included for women and data from 13 different test meals for children; the analysis included a total of 213 iron bioavailability measures in women and 232 measures in children.

The prevalence of *H. pylori* infection among women and children was 51.3% and 54.4%, respectively (Table 2). The age of the women ranged from 16 to 43 years-old, the age of the children ranged from 18 to 74 months. *H. pylori* infected women were significantly older than non-infected women (*p* < 0.05) (Table 2). All anthropometric measures (weight, height, and all z-scores in children) did not differ significantly between *H. pylori* infected and non-infected children nor women (Table 2). Iron status (Hb, SF and SF adjusted for inflammation) and inflammation (CRP) did not differ between the infected and non-infected groups. Among the *H. pylori* positive women, 27% (*n* = 11) were anemic, 22% (*n* = 9) had ID and 15% (*n* = 6) had IDA, adjusted for inflammation prevalence of ID was 41% (*n* = 17) and of IDA 22% (*n* = 9). Among the *H. pylori* negative women, 31% (*n* = 12) were anemic, 23% (*n* = 9) had ID and 21% (*n* = 8) had IDA, when adjusting for inflammation prevalence of ID was 38% (*n* = 15) and IDA 26% (*n* = 10). Among the *H. pylori* positive children, 42% (*n* = 21) were anemic, 8% (*n* = 4) had ID and 6% (*n* = 3) had IDA, and 32% (*n* = 16) had ID and 20% (*n* = 10) had IDA when adjusting for inflammation. Among the *H. pylori* negative children, 27% (*n* = 11) were anemic, 12% (*n* = 5) had ID and 5% (*n* = 2) had IDA, and 27% (*n* = 11) had ID and 15% (*n* = 6) had IDA when adjusting for inflammation.

Factors predicting fractional iron absorption in women and children are shown in Table 3. In children, the adjusted R^2^ was 0.218, predictors were: Food matrix of the test meal (*p* < 0.001); Fe compound (*p* < 0.001); whether the meal contained an iron absorption enhancer (*p* < 0.001) or inhibitor (*p* < 0.001); and the age (*p* = 0.019) (estimates and standard errors are listed in Table 3). Removed variables were: *H. pylori* infection, gender, Hb concentration and SF (adjusted for inflammation) concentration. In women the adjusted R^2^ was 0.254, variables in the minimal model were: Food matrix (*p* < 0.001); presence of iron absorption inhibitor (*p* < 0.001); SF (adjusted for inflammation) (*p* < 0.001); and Hb concentration (*p* = 0.273). Removed variables were: *H. pylori* infection, Fe compound, and age.

**Figure 2:**
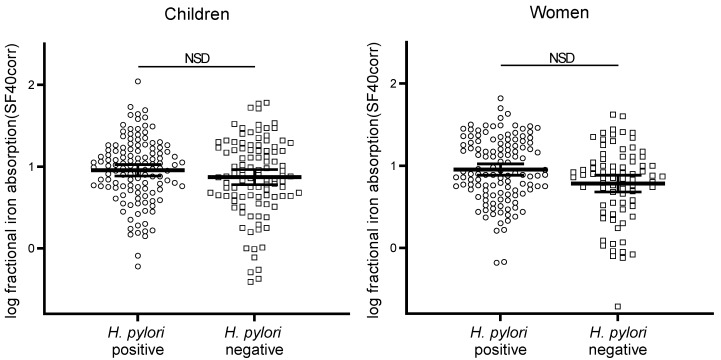
Log fractional iron absorption adjusted for SF of 40 µg/L, of *H. Pylori* positive and negative children (*n* = 90) and women (*n* = 80), each dot represents a test meal. *H. Pylori* positive children: *n* = 128, negative: *n* = 107, women *n* = 126 and *n* = 87 respectively. The line indicates the mean and 95% CI. There were no significant differences (NSD) between *H. Pylori* positive versus negative women nor children, assessed by LMM (dependent variable: Fractional iron absorption adjusted for serum ferritin; fixed factors: *H. Pylori* infection, iron compound, test meal food matrix and iron absorption enhancer or inhibitor; random factor: Subject ID number).

Figure 2 shows the fractional iron absorption data from the women and children, by group, adjusted for SF. Geometric mean (95% CI) iron absorption (%). In the *H. pylori* infected and non-infected women fractional iron absorption was 9.73% (8.24, 11.48) and 8.52% (6.99, 10.39), respectively, and did not predict absorption (*p* = 0.231) in a model including iron compound (*p* = 0.179), test meal matrix (*p* = 0.154), and whether the test meal contained an iron absorption inhibitor (*p* < 0.001). After adjusting for differences in SF, fractional iron absorption was 8.97% (7.64, 10.54) and 6.06% (4.80, 7.76), respectively. In a model including iron compound (*p* = 0.167), test meal matrix (*p* < 0.001), or whether the test meal contained an iron absorption inhibitor (*p* < 0.001), *H. pylori* infection did not result in significant prediction (*p* = 0.274), as shown in Figure 2. In children, fractional iron absorption in the *H. pylori* infected group was 9.77% (8.39, 11.4) and 8.94% (7.57, 10.6) in the non-infected group. *H. pylori* status was not a significant predictor (*p* = 0.669), when the model was controlled for the iron compound (*p* < 0.001), test meal matrix (*p* = 0.002), or whether the test meal contained an iron absorption enhancer (*p* < 0.001) or inhibitor (*p* < 0.001). After adjusting for differences in SF, fractional iron absorption was 9.02% (7.68, 10.59) and 7.44% (6.01, 9.20), respectively. When controlled for an iron compound, test meal matrix, or whether the test meal contained an iron absorption enhancer or inhibitor (for all, *p* < 0.001), *H. pylori* infection was not a significant predictor (*p* = 0.479), also shown in Figure 2.

## 4. Discussion

Our findings suggest that asymptomatic *H. pylori* infection in preschool children and young women does not have a significant effect on fractional iron absorption from iron compounds commonly used as food fortificants. Two previous studies assessed iron absorption in humans with *H. pylori* infection using iron isotope techniques, and have produced equivocal results [19,20]. This study is consistent with the previous study in Bangladeshi children [20]. In 2–5 year-old children (thirteen with *H. pylori* infection and twelve uninfected) with IDA, iron absorption from ferrous sulfate and ferrous fumarate from infant cereal was measured before and after a 14-day course of eradication treatment. There was no significant difference in iron absorption comparing *H. pylori* non-infected to *H. pylori* infected children before treatment and eradication therapy did not affect iron absorption from ferrous sulfate or ferrous fumarate [20]. The authors concluded that although gastric acid output was impaired in *H. pylori*-infected children and that treatment of *H. pylori* infection improved gastric acid output, it did not significantly influence iron absorption.

However, these study results differ from those in the study by Lopez de Romana et al. [19] in which the effect of *H. pylori* infection (assessed using the ^13^ C urea breath test) on iron absorption was compared in iron-sufficient asymptomatic adults, 24 who were *H. pylori*-positive and 26 who were *H. pylori* -negative. They consumed wheat flour-based test meals fortified with radiolabeled ferrous sulfate or ferrous fumarate. The *H. pylori*-negative subjects absorbed significantly more iron from ferrous sulfate (10.5% vs. 4.4%) and ferrous fumarate (0.6% vs. 0.4%). Iron absorption was not significantly different between groups after they received a proton pump inhibitor. Compared to the women in this study, the adults in that study had better iron status (mean serum ferritin, ≈45 µg/L versus 30 µg/L in this study) and received a much larger iron dose (55 mg) given with the test meal, compared to the smaller dosages provided in the test meals in this study (3–6 mg). Further, the diagnosis of *H. pylori* infection was made with different methods.

In a study by Ciacci et al. [18] in adults (*n* = 55) who were *H. pylori* positive or negative, serum iron levels were measured before and 2 hours after oral supplementation of 1 mg ferrous iron per kg bodyweight. *H. pylori* positive subjects were then administered antibiotic therapy, and the oral iron absorption test was repeated. They reported that *H. pylori* positive subjects before treatment had a smaller increase in serum iron compared to *H. pylori* negative subjects, and after *H. pylori* eradication in the *H. pylori* positive subjects, their serum iron increase was similar to those of non-infected subjects, suggesting that *H. pylori* infection impairs oral iron uptake [18]. Sarker et al. [21] randomized *H. pylori* infected children 2–5 years of age with IDA to receive 2-week anti-*H. pylori* therapy plus 90-day oral ferrous sulfate, 2-week anti-*H. pylori* therapy alone, 90-day oral iron alone, or placebo; non-infected children with IDA received iron treatment as a negative control. *H. pylori* infection did not inhibit the response to iron, suggesting it is not a cause of iron deficiency or a reason for treatment failure of iron supplementation in this setting [21]. However, other studies have suggested that *H. pylori* infected children show a blunted response to oral iron [39]. A recent systematic review reported that, in observational studies, compared to uninfected persons, *H. pylori* infected individuals are at greater risk for iron deficiency and iron deficiency anemia. Also, prospective trials comparing *H. pylori* eradication therapy plus iron supplementation, as compared with iron supplementation alone, showed greater increases in serum ferritin with combined therapy [8].

Several mechanisms have been suggested as a potential cause of iron deficiency and/or low iron absorption during *H. pylori* infection. Chronic gastritis, due to *H. pylori*, can alter the physiology of the stomach by reducing gastric acid secretion and gastric ascorbic acid levels. Both of which are essential for the absorption of dietary iron [11,12,13,14,15,16,17]. *H. pylori* requires iron for its growth [40,41], it expresses proteins associated with iron metabolism [41], and it is suggested that it may disrupt host hepcidin regulation [42]. A decrease in serum hepcidin levels has been reported after an *H. pylori* eradication therapy, in two studies [43,44], however one of them concluded that the decrease is more related to anemia status than to *H. pylori* infection as a decrease of hepcidin was also reported in the group receiving iron supplement only without *H. pylori* eradication therapy [44]. Finally, iron losses may increase due to occult bleeding from *H. pylori* gastritis [42,45]. Our finding that asymptomatic *H. pylori* infection does not impair fractional iron absorption from a meal, suggests that if asymptomatic *H. pylori* infection increases the risk for iron deficiency [8], it may do this through increasing gastrointestinal blood loss, rather than reducing dietary iron absorption.

The model looking at predictors of iron absorption from fortified test meals confirms earlier studies which showed that food composition, or the addition of ascorbic acid, phytase or a polyphenol-rich tea, affect iron absorption in both women and children [23,46]. Iron status is a commonly known predictor of iron absorption [38,47], and the current study data in women confirm this. In contrast, the current data in preschool children suggest that iron status does not predict iron absorption; however, this can potentially be explained by the narrow range of SF values in this group.

Both the severity and location of *H. pylori* related gastritis might be determinants of gastric function and acidity, and iron absorption. Gastritis affecting the antral mucosa increases the release of gastrin and secretion of acid. In contrast, when gastritis is predominant in the corpus this impairs parietal cell acid secretion, leading to hypochlorhydria, this form of infection is seen in most infected subjects [10]. *H. pylori* positive children with hypochlorhydria (pH > 4) were hypoferremic compared to *H. pylori* positive children with gastric pH ≤ 4 [16]. When gastritis affects both the antrum and corpus, the acid output may remain normal [10]. Altogether, these data suggest that *H. pylori* gastritis may alter gastric physiology to favor either an increase or a decrease in absorption of dietary iron, and this may confound studies, such as the present study, looking at differences in iron absorption-based only on serology. However, the location of gastric inflammation caused by *H. pylori* can be determined only with endoscopy, which we did not perform in our subjects.

Our study has several strengths. It is the first pooled analysis to date assessing the association of asymptomatic *H. pylori* infection with fractional iron absorption quantified using iron stable isotopes. We studied two risk groups for iron deficiency, young women and preschool children, residing in three low-income countries. We were able to assess potential effects from a diverse range of test meals and three commonly used iron fortificants. We used linear mixed model analysis to assess whether *H. pylori* infection predicted fractional iron absorption, controlling for age, hemoglobin, iron, inflammation status, and test meal matrix, all measured using standardized methods across the studies. However, there are several limitations to this study. It was retrospective, and assessment of *H. pylori* infection was performed using serology, which may produce false positives results, because (despite an eradicated infection) IgG titers may remain in the serum and decline only over several months [48]. However, none of the subjects in this study had received recent *H. pylori* eradication therapy. A more precise assessment method of a current *H. pylori* infection would have been a ^13^ C urea breath test. Also, the assessment of gastric acid output before and after *H. pylori* eradication would have been valuable to distinguish between antral and corpus infection. Despite these limitations, this study provides important new data on the link between *H. pylori* and iron absorption, and suggests, in asymptomatic women and preschool children in low-income settings, *H. pylori* status is not a major predictor of fractional iron absorption from iron fortificants.

## Figures and Tables

**Table 1:** Overview of the studies included in this analysis: Study population, sample size, test meals and iron compounds given.

Study	Location	Age Group	N	Test Meal Matrix	Fe/Meal (mg)	Study Arms (Fe Compound, Inhibitor or Enhancer)
1	Benin	Preschool children	18	Pearl millet porridge	6	1. FeSO4
						2. FeSO4 and NaFeEDTA ^1^
2	Benin	Preschool children	16	Pearl millet porridge	6	1. FeSO_4_
						2. FeSO_4_ and Ascorbic Acid
3	Benin	Preschool children	18	Pearl millet porridge	6	1. FeSO_4_
						2. FeSO_4_ and phytase
						3. FeSO_4_ and phytase and Ascorbic Acid
4	Senegal	Preschool children	17	Wheat bread	2	1. Fe Fumarate
						2. FeSO_4_
						3. Fe Fumarate and tea infusion
						4. FeSO_4_ and tea infusion
5	Haiti	Preschool children	21	Wheat bread	2	1. Fe Fumarate
						2. NaFeEDTA
6	Benin	Women	23	Fermented sorghum porridge	3	1. NaFeEDTA
7	Benin	Women	20	Pearl millet paste	4	1. Regular millet: FeSO_4_
						2. Biofortified millet: FeSO_4_
						3. Post-harvest fortified millet: FeSO_4_
8	Senegal	Women	17	Wheat bread	4	1. Fe Fumarate
						2. FeSO_4_
						3. Fe Fumarate and tea infusion
						4. FeSO_4_ and tea infusion
9	Haiti	Women	21	Wheat bread	4	1. Fe Fumarate
						2. NaFeEDTA
						3. NaFeEDTA and Fe fumarate ^2^

^1^ 3 mg Fe as FeSO_4_ mixed with 3 mg Fe as NaFeEDTA. ^2^ 2 mg Fe as NaFeEDTA mixed with 2 mg Fe fumarate.

**Table 2:** Subject characteristics: Age, anthropometrics, hemoglobin (Hb), serum ferritin (SF), and C-reactive protein (CRP) concentrations, of all subjects grouped by *H. pylori* infection status and age group ^1^.

	Children			Women		
*H. pylori* Status	Positive	Negative	*p*	Positive	Negative	*p*
n	49	41	-	41	39	-
Age y	2.8 (2.6, 3.1) ^2^	2.9 (2.6, 3.2)	0.882	25.7 (23.4, 28.3)	21.8 (20.2, 23.6)	0.007
Weight, kg	12.3 (11.7, 13.0)	12.4 (11.7, 13.2)	0.975	55.1 (53.3, 57.0)	55.0 (52.7, 57.4)	0.997
Height, cm	91 (89, 94)	92 (89, 95)	0.972	160 (158, 162)	162 (159, 164)	0.447
WAZ	−0.94 ± 0.78 ^3^	−0.93 ± 1.08	0.759	n.a.		-
HAZ	−0.88 ± 1.32	−0.89 ± 1.63	0.818	n.a.		-
WHZ	−0.68 ± 0.87	−0.67 ± 1.10	0.938	n.a.		-
Hb g/L	109 (107, 112)	114 (110, 117)	0.052	126 (121, 132)	125 (121, 130)	0.776
SF µg/L	32.6 (26.7, 39.8)	31.7 (25.1, 40.0)	0.855	30.1 (22.1, 41.0)	30.0 (22.9, 39.4)	0.989
SF adjusted ^4^ µg/L	15.8 (12.9, 19.4)	16.6 (13.3, 20.7)	0.744	18.2 (13.8, 24.1)	19.1 (14.6, 25.1)	0.797
CRP mg/L	1.20 (0.71, 2.03)	0.82 (0.49, 1.37)	0.499	0.67 (0.43, 1.05)	0.60 (0.41, 0.88)	0.696
CRP > 5 mg/L (n)	12	8	-	2	3	-

^1^ Differences between *H. pylori* positive versus negative were assessed by unpaired *t*-test. WAZ, weight-for-age z score; HAZ, height-for-age z score; WHZ, weight-for-height z score; n.a., not applicable; Hb, hemoglobin; SF, serum ferritin; CRP, C-reactive protein. ^2^ All such values are geometric means (95% CIs). ^3^ All such values are means ± SD. ^4^ Serum ferritin adjusted for C-reactive protein [37].

**Table 3:** The minimal adequate model predicting fractional iron absorption of preschool children (*n* = 90) and women of reproductive age (*n* = 80) in relation to *H. pylroi* infection, food matrix, iron compound, whether the test meal contained an iron absorption enhancer or inhibitor, gender, age, hemoglobin and serum ferritin adjusted for inflammation ^1^.

	Children ^2^			Women ^3^		
Variables	*b*	SE	*p*	*b*	SE	*p*
Intercept	1.62	0.21	0.000	−0.22	1.69	0.898
*H. pylori* infection	removed from the model **^4^**			removed from the model		
Food Matrix (all pairwise)						
wheat bread-millet paste		n.a.		0.34	0.09	0.000
wheat bread-fermented sorghum		n.a.		−0.21	0.09	0.016
millet paste-fermented sorghum		n.a.		−0.55	0.10	0.000
millet-porridge-wheat bread	0.41	0.11	0.000		n.a.	
Fe compound (all pairwise)				removed from the model		
Sulfate-Fumarate	0.17	0.05	0.001			
Sulfate-EDTA	0.05	0.08	0.557			
Sulfate-Sulfate and EDTA	0.15	0.06	0.024			
Fumarate-EDTA	−0.13	0.07	0.055			
Fumarate-Sulfate and EDTA	−0.02	0.08	0.762			
EDTA-Sulfate and EDTA	0.10	0.10	0.320			
Fe absorption enhancer					n.a.	
Ascorbic Acid-none	0.19	0.07	0.005			
Phytase-none	0.29	0.06	0.000			
Ascorbic Acid and Phytase-none	0.36	0.06	0.000			
Fe absorption inhibitor						
Tea-none	−0.42	0.05	0.000	−0.33	0.05	0.000
Gender	removed from the model				n.a.	
Age	−0.83	0.34	0.019	removed from the model		
Hemoglobin	removed			0.93	0.85	0.273
Serum ferritin adjusted for CRP	removed			−0.52	0.13	0.000

^1^ The minimal adequate model assessed by backward regression. Estimates (*b*) and standard errors (SE) assessed by linear mixed model: Random factor: Subject ID number; dependent variable: Fractional iron absorption; fixed factors in children’s model: Food matrix, Fe compound, Fe absorption enhancer, Fe absorption inhibitor, and age; fixed factors in women’s model: Food matrix, Fe absorption inhibitor, hemoglobin and serum ferritin adjusted for CRP (C-reactive protein). ^2^ Minimal adequate regression model of children: *R*^2^ = 0.239; adjusted *R*^2^ = 0.218. ^3^ Minimal adequate regression model of women: *R*^2^ = 0.272; adjusted *R*^2^ = 0.254. ^4^ Removed variable by the backward regression to assess the minimal adequate model.

## References

[1] Blaser M.J., Atherton J.C. (2004). *Helicobacter pylori* persistence: Biology and disease. J. Clin. Investig..

[2] Atherton J.C. (2006). The pathogenesis of *Helicobacter pylori*-induced gastro-duodenal diseases. Annu. Rev. Pathol..

[3] Wongphutorn P., Chomvarin C., Sripa B., Namwat W., Faksri K. (2018). Detection and genotyping of *Helicobacter pylori* in saliva versus stool samples from asymptomatic individuals in Northeastern Thailand reveals intra-host tissue-specific *H. pylori* subtypes. BMC Microbiol..

[4] Mathewos B., Moges B., Dagnew M. (2013). Seroprevalence and trend of *Helicobacter pylori* infection in Gondar University Hospital among dyspeptic patients, Gondar, North West Ethiopia. BMC Res. Notes.

[5] Kienesberger S., Perez-Perez G.I., Olivares A.Z., Bardhan P., Sarker S.A., Hasan K.Z., Sack R.B., Blaser M.J. (2018). When is *Helicobacter pylori* acquired in populations in developing countries? A birth-cohort study in Bangladeshi children. Gut Microbes.

[6] Sarker S.A., Mahalanabis D., Hildebrand P., Rahaman M.M., Bardhan P.K., Fuchs G., Beglinger C., Gyr K. (1997). *Helicobacter pylori*: Prevalence, transmission, and serum pepsinogen II concentrations in children of a poor periurban community in Bangladesh. Clin. Infect. Dis..

[7] Nahar S., Kaderi Kibria K.M., Hossain M.E., Sarker S.A., Bardhan P.K., Talukder K.A., Rahman M. (2018). Epidemiology of *H. pylori* and its Relation with Gastrointestinal Disorders, A Community-based Study in Dhaka, Bangladesh. J. Gastroenterol. Hepatol. Res..

[8] Hudak L., Jaraisy A., Haj S., Muhsen K. (2017). An updated systematic review and meta-analysis on the association between *Helicobacter pylori* infection and iron deficiency anemia. Helicobacter.

[9] Xu M.Y., Cao B., Yuan B.S., Yin J., Liu L., Lu Q.B. (2017). Association of anaemia with *Helicobacter pylori* infection: A retrospective study. Sci. Rep..

[10] McColl K.E., el-Omar E., Gillen D. (1998). Interactions between *H. pylori* infection, gastric acid secretion and anti-secretory therapy. Br. Med. Bull..

[11] Calam J., Gibbons A., Healey Z.V., Bliss P., Arebi N. (1997). How does *Helicobacter pylori* cause mucosal damage? Its effect on acid and gastrin physiology. Gastroenterology.

[12] Zhang Z.W., Patchett S.E., Perrett D., Katelaris P.H., Domizio P., Farthing M.J. (1998). The relation between gastric vitamin C concentrations, mucosal histology, and CagA seropositivity in the human stomach. Gut.

[13] Nahon S., Lahmek P., Massard J., Lesgourgues B., de Serre N.M., Traissac L., Bodiguel V., Adotti F., Delas N. (2003). *Helicobacter pylori*-associated chronic gastritis and unexplained iron deficiency anemia: A reliable association?. Helicobacter.

[14] Kaye P.V., Garsed K., Ragunath K., Jawhari A., Pick B., Atherton J.C. (2008). The clinical utility and diagnostic yield of routine gastric biopsies in the investigation of iron deficiency anemia: A case-control study. Am. J. Gastroenterol..

[15] Lee S.Y., Yang J.H., Hong S.N., Kim J.H., Sung I.K., Park H.S., Shim C.S. (2012). Low Hemoglobin Levels are Related to the Presence of Gastric Atrophy Rather Than the Presence of *H. pylori* Infection Itself: A Study of 2398 Asymptomatic Adults. Gastroenterology.

[16] Harris P.R., Serrano C.A., Villagran A., Walker M.M., Thomson M., Duarte I., Windle H.J., Crabtree J.E. (2013). *Helicobacter pylori*-associated hypochlorhydria in children, and development of iron deficiency. J. Clin. Pathol..

[17] Queiroz D.M., Harris P.R., Sanderson I.R., Windle H.J., Walker M.M., Rocha A.M., Rocha G.A., Carvalho S.D., Bittencourt P.F., de Castro L.P. (2013). Iron status and *Helicobacter pylori* infection in symptomatic children: An international multi-centered study. PLoS ONE.

[18] Ciacci C., Sabbatini F., Cavallaro R., Castiglione F., di Bella S., Iovino P., Palumbo A., Tortora R., Amoruso D., Mazzacca G. (2004). *Helicobacter pylori* impairs iron absorption in infected individuals. Dig. Liver Dis..

[19] Lopez de Romana D., Pizarro F., Diazgranados D., Barba A., Olivares M., Brunser O. (2011). Effect of *Helicobacter pylori* infection on iron absorption in asymptomatic adults consuming wheat flour fortified with iron and zinc. Biol. Trace Elem. Res..

[20] Sarker S.A., Davidsson L., Mahmud H., Walczyk T., Hurrell R.F., Gyr N., Fuchs G.J. (2004). *Helicobacter pylori* infection, iron absorption, and gastric acid secretion in Bangladeshi children. Am. J. Clin. Nutr..

[21] Sarker S.A., Mahmud H., Davidsson L., Alam N.H., Ahmed T., Alam N., Salam M.A., Beglinger C., Gyr N., Fuchs G.J. (2008). Causal relationship of *Helicobacter pylori* with iron-deficiency anemia or failure of iron supplementation in children. Gastroenterology.

[22] Lopez A., Cacoub P., Macdougall I.C., Peyrin-Biroulet L. (2015). Iron deficiency anaemia. Lancet.

[23] Allen L., World Health Organization (2006). Guidelines on Food Fortification with Micronutrients. Bruno De Benoist, Omar Dary, Richard Hurrell.

[24] Hurrell R.F. (2015). Flour fortification as a strategy to prevent anaemia. Br. J. Nutr..

[25] World Health Organization (2009). Recommendations on Wheat and Maize Flour Fortification Meeting Report: Interim Consensus Statement.

[26] Food Fortification Initiative (2018). Global Progress of Indistrually Milled Cereal Grains. http://www.ffinetwork.org/global_progress/index.php.

[27] Cercamondi C.I., Egli I.M., Ahouandjinou E., Dossa R., Zeder C., Salami L., Tjalsma H., Wiegerinck E., Tanno T., Hurrell R.F. (2010). Afebrile Plasmodium falciparum parasitemia decreases absorption of fortification iron but does not affect systemic iron utilization: A double stable-isotope study in young Beninese women. Am. J. Clin. Nutr..

[28] Cercamondi C.I., Egli I.M., Mitchikpe E., Tossou F., Hessou J., Zeder C., Hounhouigan J.D., Hurrell R.F. (2013). Iron bioavailability from a lipid-based complementary food fortificant mixed with millet porridge can be optimized by adding phytase and ascorbic acid but not by using a mixture of ferrous sulfate and sodium iron EDTA. J. Nutr..

[29] Cercamondi C.I., Egli I.M., Mitchikpe E., Tossou F., Zeder C., Hounhouigan J.D., Hurrell R.F. (2013). Total iron absorption by young women from iron-biofortified pearl millet composite meals is double that from regular millet meals but less than that from post-harvest iron-fortified millet meals. J. Nutr..

[30] Herter-Aeberli I., Eliancy K., Rathon Y., Loechl C.U., Pierre J.M., Zimmermann M.B. (2017). In Haitian women and preschool children, iron absorption from wheat flour-based meals fortified with sodium iron EDTA is higher than that from meals fortified with ferrous fumarate, and is not affected by Helicobacter pylori infection in children. Br. J. Nutr..

[31] World Health Organization (2001). Iron Deficiency Anaemia: Assessment, Prevention and Control: A Guide for Programme Managers.

[32] Rapid Labs (2014). H. pylori Ab Rapid Test, Manufactures Instructions.

[33] Brown E., Bradley B., Wennesland R., Hodges J.L., Hopper J., Yamauchi H. (1962). Red Cell, Plasma, and Blood Volume in Healthy Women Measured by Radichromium Cell-Labeling and Hematocrit. J. Clin. Investig..

[34] Walczyk T., Davidsson L., Zavaleta N., Hurrell R.F. (1997). Stable isotope labels as a tool to determine the iron absorption by Peruvian school children from a breakfast meal. Fresenius J. Anal. Chem..

[35] Turnlund J.R., Keyes W.R., Peiffer G.L. (1993). Isotope ratios of molybdenum determined by thermal ionization mass spectrometry for stable isotope studies of molybdenum metabolism in humans. Anal. Chem..

[36] International Atomic Energy Agency (2012). Assessment of Iron Bioavailability in Humans Using Stable Iron Isotope Techniques.

[37] Namaste S.M., Rohner F., Huang J., Bhushan N.L., Flores-Ayala R., Kupka R., Mei Z., Rawat R., Williams A.M., Raiten D.J. (2017). Adjusting ferritin concentrations for inflammation: Biomarkers Reflecting Inflammation and Nutritional Determinants of Anemia (BRINDA) project. Am. J. Clin. Nutr..

[38] Cook J.D., Dassenko S.A., Lynch S.R. (1991). Assessment of the role of nonheme-iron availability in iron balance. Am. J. Clin. Nutr..

[39] Duque X., Moran S., Mera R., Medina M., Martinez H., Mendoza M.E., Torres J., Correa P. (2010). Effect of eradication of *Helicobacter pylori* and iron supplementation on the iron status of children with iron deficiency. Arch. Med. Res..

[40] Otto B.R., van Vught Verweij A.M., MacLaren D.M. (1992). Transferrins and heme-compounds as iron sources for pathogenic bacteria. Crit. Rev. Microbiol..

[41] van Vliet A.H., Stoof J., Vlasblom R., Wainwright S.A., Hughes N.J., Kelly D.J., Bereswill S., Bijlsma J.J., Hoogenboezem T., Vandenbroucke-Grauls C.M. (2002). The role of the Ferric Uptake Regulator (Fur) in regulation of *Helicobacter pylori* iron uptake. Helicobacter.

[42] Beutler E. (2007). Hepcidin mimetics from microorganisms? A possible explanation for the effect of *Helicobacter pylori* on iron homeostasis. Blood Cells Mol. Dis..

[43] Sapmaz F., Basyigit S., Kalkan I.H., Kisa U., Kavak E.E., Guliter S. (2016). The impact of *Helicobacter pylori* eradication on serum hepcidin-25 level and iron parameters in patients with iron deficiency anemia. Wien. Klin. Wochenschr..

[44] Lee S.Y., Song E.Y., Yun Y.M., Yoon S.Y., Cho Y.H., Kim S.Y., Lee M.H. (2010). Serum prohepcidin levels in *Helicobacter pylori* infected patients with iron deficiency anemia. Korean J. Intern. Med..

[45] Yip R., Limburg P.J., Ahlquist D.A., Carpenter H.A., O’Neill A., Kruse D., Stitham S., Gold B.D., Gunter E.W., Looker A.C. (1997). Pervasive occult gastrointestinal bleeding in an Alaska native population with prevalent iron deficiency. Role of *Helicobacter pylori* gastritis. JAMA.

[46] World Health Organization (2005). Vitamin and Mineral Requirements in Human Nutrition.

[47] Cook J.D., Lipschitz D.A., Miles L.E., Finch C.A. (1974). Serum ferritin as a measure of iron stores in normal subjects. Am. J. Clin. Nutr..

[48] Urita Y., Hike K., Torii N., Kikuchi Y., Kurakata H., Kanda E., Sasajima M., Miki K. (2004). Comparison of serum IgA and IgG antibodies for detecting *Helicobacter pylori* infection. Intern. Med..

